# Evaluating the probiotic and therapeutic potentials of *Saccharomyces cerevisiae* strain (OBS2) isolated from fermented nectar of toddy palm

**DOI:** 10.1186/s13568-016-0301-1

**Published:** 2017-01-03

**Authors:** Banoth Srinivas, Ganapathiwar Swarupa Rani, Bhukya Kiran Kumar, Banoth Chandrasekhar, Kommalapati Vamsi Krishna, Tangutur Anjana Devi, Bhukya Bhima

**Affiliations:** 1 Department of Microbiology, University College of Science, Osmania University, Hyderabad, Telangana 500007 India; 2 Center for Chemical Biology, CSIR-Indian Institute of Chemical Technology, Tarnaka, Hyderabad, Telangana 500007 India

**Keywords:** Probiotics, Toddy nectar, Antimicrobial resistance, Therapeutics, Yeasts

## Abstract

The purpose of this study is to evaluate the probiotic characteristics of 15 yeast strains isolated from nectar of toddy palm. Initially, the collected samples were inoculated on yeast extract peptone dextrose agar plates and the colonies so obtained were culturally and morphologically characterized. Commercial probiotic yeast, *Saccharomyces boulardii* served as the control in these experiments. Of the 15 yeast strains, the isolates that were resistant to antibiotics and worked synergistically with other cultures were considered for further evaluation. Selected isolates were evaluated in vitro for tolerance to simulated gastrointestinal conditions such as temperature, pH, bile and gastric juice. Further the yeast isolates were evaluated for their pathogenicity and adherence to intestinal epithelial cells. The 2 yeast isolates with efficient probiotic properties were finally characterized by sequencing their 5.8 S rRNA and partial sequences of internal transcribed spacer 1 and 2. The sequences were BLAST searched in the National Center for Biotechnology Information, nucleic acid database for sequence similarity of organisms and phylogenetic evolutionary analysis was carried out. Based on maximum similarity of basic local alignment search tool results, organisms were characterized as *Pichia kudriavzevii* OBS1 (100%) and *Saccharomyces cerevisiae* OBS2 (96%) and sequences were finally deposited in the GenBank data library. Among these two isolates, *S. cerevisiae* OBS2 displayed slight/moderate antioxidant and anticancer property. Hence, strain OBS2 can be utilized and explored as a potential probiotic for therapeutic applications.

## Introduction

It is well known that many types of bacteria and yeast strains are being used to enhance the fermentative reactions in food industries including bread, beer, wine and xylitol (Ortiz et al. 2013). Besides fermentation, these organisms are playing numerous beneficial roles in human, animal health and nutrition. A number of previous studies reported about the use of probiotics for the prevention and treatment of gastrointestinal infections including food borne pathogens associated infections (Fooks and Gibson 2002). In addition, it is reported that yeast promotes both human and animal health (Zanello et al. 2009; O’Neil et al. 2012) and enhances the bioavailability of minerals through hydrolysis of phytate, folate biofortification, detoxification of mycotoxins and xenobiotics (Ohashi and Kazunari 2009). At present, the products of wild and recombinant yeast form the backbone for many commercially important sectors, including foods, beverages, pharmaceuticals, industrial enzymes and medicine (Mc Meekin et al. 1997). *Saccharomyces cerevisiae* has a Qualified Presumption of Safety (QPS) status (Verna and Lucak 2010; Waters et al. 2012). Potential probiotic mechanism of *S. cerevisiae* is based on emission of inhibitory proteins against pathogens, stimulation of host immunoglobulin A and exclusion of secreted toxins in host intestine (Fooks and Gibson 2002).

Tolerance to low pH, high temperature, high ox-bile concentration, a mixture of organic acids concentration, digestive enzymes like amylase, pepsin and trypsin concentrations and ability to produce several other useful metabolites represent the beneficial characteristics of probiotics (Fridkin and Jarvis 1996). Probiotics need to survive the inevitable biological barriers of the gut. It is evidently proven from the previous in vitro studies that eukaryotic probiotics can tolerate the well simulated environmental conditions of the digestive tract (Kumura et al. 2004). The probiotic strains of *S. cerevisiae* are not only able to survive in the digestive tract of the host, but also found to persist at high populations (Blehaut et al. 1989). The pharmacokinetic studies indicate that the probiotic *Sachcharomyces var boulardii* when ingested orally achieved a steady state in the gut within 3 days, but eliminated within 2–5 days after it is discontinued. Such elimination from the digestive tract is believed to be due to the barrier effect of the complex established by resident microbiota of the gut (Fridkin and Jarvis 1996; Yoo and Kim 2016).

Yeast with its derivatives or by-products has gained huge importance and become a model organism for many biological functions in our life. *S. cerevisiae* is a potential organism to be explored as novel natural anticancer, antioxidant and immunostimulating agent for using in functional foods or medicine (Hassan 2011; Balasubramanian and Ragunathan 2012).

Since the droppings and exudates of toddy palm during hot summer stimulate the growth of simple sugar fermenting, high temperature (~40 °C) resistant microorganisms (Abdel Fattah et al. 2000), in the present study, yeasts were isolated from fermented nectar of toddy palm. The isolated yeast samples were subjected to a series of analyses for efficient probiotic and therapeutic characteristics.

## Materials and methods

### Isolation of yeast strains

Samples were collected from naturally fermented nectar of toddy palm during mid day of hot summer (~40 °C) from different regions of Telangana State, India. Commercial probiotic yeast *S. boulardii* (Econorm, Dr. Reddy’s Laboratories, Hyderabad, India) was used as a referral organism.

Isolation of yeasts was carried out by using standard methodology described by Tikka et al. (2013) and Zaky et al. (2014). After incubation, colonies were selected based on morphology and sub-cultured on yeast extract peptone dextrose (YPD) agar slants for further observations.

### Growth and characterization of yeast isolates

Isolates were characterized by their cultural and morphological characteristics following the protocols as described by Reis et al. (2013). After incubation, cultural and morphological characteristics were observed by visible growth pattern followed by simple staining and microscopic observation.

The sugar utilization ability of yeast isolates was achieved by their sugar assimilation profiling. The sugar assimilation property enables us to determine the ability of yeast to use a specific sugar/carbohydrate as a sole source of carbon. Yeast nitrogen base (YNB) medium with 2% (w/v) specific selected sugar (s) like glucose, maltose, ribose, fructose, xylose, cellobiose, dextrose, mannose, starch, sucrose, lactose and galactose were used for determination of yeast sugar assimilation property on the basis of biomass formation (Marinho et al. 2010).

### Assessment for probiotic properties

#### Antimicrobial drug sensitivity

The yeast isolates were tested for antimicrobial drug sensitivity against 8 antifungal agents like Cefmetazole 30 µg (CMZ 30), Ceftazidime 10 µg (CTR 10), Clotrimazole 10 µg (CC 10), Fluconazole 10 µg (FLC 10), Itraconazole 10 µg (IT 10), Ketoconazole 50 µg (KT 50), Miconazole 50 µg (MIC 50) and Nystatin 100 µg (NS 100) and 20 antibacterial agents like Amoxicillin 10 µg (AMX 10), Ampicillin 10 µg (AMP 10), Ampicillin 25 µg (AMP 25), Bacitracin 10 µg (B 10), Ceftriaxone 10 µg (CT 10), Chloramphenicol 10 µg (C 10), Clindamycin 10 µg (CD 10), Cloxacillin 10 µg (COX 10), Enrofloxacin 10 µg (EX 10), Erythromycin 10 µg (E 10), Gentamicin 10 µg (GEN 10), Kanamycin 30 µg (K 30), Lincomycin 10 µg (L 10), Methicillin 10 µg (MET 10), Neomycin 10 µg (N 10), Norfloxacin 10 µg (NX 10), Penicillin G 10 µg (P 10), Streptomycin 10 µg (S 10), Streptomycin 25 µg (S 25), Teracycline 10 µg (TE 10), Teracycline 30 µg (TE 30), Trimethoprim 10 µg (TR 10) and Vancomycin 10 µg (VA 10) of Sigma Aldrich (Sigma Aldrich Pvt. Ltd., India) were placed on yeast inoculated YPD medium plates and incubated for 48 h at 30 °C. After incubation drug sensitivity of the yeast isolates was evaluated by measuring the zone of inhibition (Mashad et al. 2012).

#### Antagonistic activity against pathogens

Antagonistic activity of yeast isolates was evaluated by agar well plate method by measuring the zone of inhibition around the wells as per protocol described by Sibanda et al. (2010) against human intestinal pathogenic bacteria like *Escherichia coli* 0157:H7, *Pseudomonas aeruginosa*, *Klebsiella pneumonia*, *Staphylococcus aureus*, *Salmonella typhi* and *Salmonella paratyphi.*

#### Co-culture activity with healthy commensals

Co-culture activity assay is yet another method to determine the positive or negative impact and/or ability of candidate probiotic yeast isolates to coexist with normal bacterial flora of the human intestine. Yeast isolates and bacterial cultures like *E*. *coli* 0150:H5, *Lactobacillus acidophilus* were activated in their basal media and equal volumes (0.1 ml) of their suspensions were inoculated in a modified medium (1% yeast extract, 1% peptone, 0.25% NaCl and 1% dextrose) with pH 6.5 and incubated at 37 °C for 24 h. After incubation, culture suspensions were diluted to tenfold and 10 µl of 10^−3^ dilution was inoculated on YPD agar medium with Ampicillin (30 µg/ml), Kanamycin (30 µg/ml) for yeast growth and nutrient agar medium with Geneticin (200 µg/ml) for bacterial growth. The YPD agar medium plates were incubated at 30 °C for 48 h and nutrient agar plates were incubated at 37 °C for 24 h. After incubation, percent viability of yeast isolates and bacteria was determined on the basis of colony forming units (CFU) using modified methodology of Paschos et al. (2015).

#### Stress tolerance

A crucial step towards the identification and selection of potential probiotic candidates is to evaluate their resistance to the extreme conditions of the gastrointestinal tract. The barriers that must be overcome are temperature, low pH, organic acids and digestive enzymes, i.e. pepsin and amylase in the stomach and trypsin and bile in the upper intestine (Corzo and Gilliland 1999). The probiotic organism should be able to tolerate high temperature, i.e. above 37 °C and the diverse conditions of the gastric juice. Therefore, to test the parameters of stress tolerance of yeast isolates the following experiments were performed.

#### Resilience to gastrointestinal parameters

Actively growing yeast suspensions (1 ml) were inoculated in 100 ml phosphate buffered saline (PBS) and incubated for 4 h at different temperatures (30, 37, 40 and 45 °C), with different pH (2.0, 2.5, 3.0 and 3.5), different percentages of ox bile (0.25, 0.5, 0.75 and 1.0) and different percent mixture of 3 organic acids like propionic, butyric and acetic acids in 7:2:1 ratio (0.25, 0.5, 0.75 and 1.0). After incubation, 100 µl of inoculum from each set was inoculated on YPD agar at 30 °C for 24 h and percent tolerance was calculated by using the formula (*log N*/*log N*_*0*_) × 100 where N is count after incubation and N_0_ is count before incubation (both expressed as cfu/ml) (Syal and Vohra 2013).

Aqueous solution (1 ml) of 5% NaCl was taken to create an in vitro gastric environment with different concentrations of enzymes like Pepsin (2.0, 4.0, and 6.0 g l^−1^), Trypsin (0.25, 0.5 and 0.75 mg ml^−1^) and Amylase (250, 300 and 350 IU ml^−1^) (Pennacchia et al. 2008). The pH of pepsin containing solution was adjusted to 2.0 and trypsin and amylase containing solution was adjusted to 7.5. All the sets were inoculated with 0.1% inoculum of yeast isolates and incubated at 30 °C in a shaking incubator at 150 rpm for 4 h. After incubation of all the sets, 100 µl of inoculum from each set was inoculated on YPD agar medium for the determination of percent tolerance of yeast isolates (Syal and Vohra 2013).

#### Pathogenicity of yeast isolates

Yeast such as *S. boulardii* are best studied probiotics being used successfully and well known to play a crucial role in the treatment and management of diarrhoea and inflammatory bowel disease and to reduce the duration of disease. Despite an excellent record of safe use, yeasts may still be the cause of localized infections in some patients.

Therefore, in order to determine whether the yeast isolates show pathogenicity, it was studied by the detection of enzymes like protease, phospholipase, the ability of haemolysis and by detection of specific pathogenic genes through polymerase chain reaction (PCR) amplification. Protease activity was tested using milk casein and gelatin as substrates by the method described by Lechuga et al. (2016) and was adapted to verify the phospholipase activity. Similarly, haemolytic activity was determined by a modified method of Manns et al. (1994). Protease and phospholipase production was detected by halos formation around the culture and haemolysin activity was detected by measurement of haemolytic index (Luo et al. 2001). Extracted genomic DNA of each isolate was amplified with universal primer SR6R (5′-AAGTAAAGT-CGTAACAAGG-3′) and LR1 (5′-GGTTGGTTTCTTTTCCT-3′) of human pathogenic *Candida albicans*. The reaction mixture for PCR and conditions are as per protocol described by Koehn (1982). The amplicons formed as PCR product were visualized on an agarose gel.

#### Cell adhesion

Caco-2 cells derived from human intestinal epithelial cells were purchased from National Centre for Cell Science (NCCS), Pune, India and maintained in Eagle’s minimal essential medium (MEM) with glutamine (0.584 g/l), sodium bicarbonate (3.7 g/l), penicillin (100 U/ml), streptomycin (100 µg/ml), and 5% serum and the final pH of medium was adjusted to 7.2 before sterilization. Culture flasks with medium were maintained in a CO_2_ incubator at 37 °C and 5% CO_2_ as per the protocol described in Nikolic et al. (2012). Inoculated monolayers were washed thrice with warm MEM medium without serum. Adhesion of yeast cells with monolayer was fixed with cold methanol, stained with Giemsa stain and observed under microscope for adherence. The mean number of yeast cells was determined and the adherence score was expressed for each yeast isolate.

### Molecular characterization

Based on the probiotic characteristics, 2 yeast isolates OBS1 and OBS2 were selected and characterized by sequencing of 5.8S rRNA and internal transcribed spacer (ITS) 1 and 2 (Fujita et al. 2001). Genomic DNA was extracted using an Insta Gene Matrix (BIO RAD, California, USA). The universal primers used for amplification of 5.8S rRNA, ITS1and ITS2 were 5′-TCCGTAGGTGAACCTGCGG-3′ and 5′-TCCTCCGCTTATTGATATGC-3′. The PCR conditions were set to 1 min each for denaturation at 95 °C, annealing at 55 °C, 2 min for an extension at 72 °C and finally finishing with a 10 min step at 72 °C. The amplicons were purified with a multiscreen filter plate (Millipore Corp., Bedford, MA, USA) and sequencing was performed using PRISM BigDye Terminator v3.1 Cycle sequencing Kit (Applied Biosystems, California, USA). The amplicons were added to Hi-Di formamide (Applied Biosystems, Foster City, CA) and the mixture was incubated at 95 °C for 5 min, followed by incubation on ice for 5 min and then analyzed by ABI Prism 3730XL DNA analyzer (Applied Biosystems). Complete sequence of 5.8S rRNA and partial sequences of internal transcribed spacer (ITS) 1 and 2 were BLAST searched in NCBI database (www.ncbi.nlm.nih.gov) for similarity. Based on maximum similarity of BLAST results, a phylogenetic dendrogram was constructed using Phylip 3.69.

### Anticancer properties

Three test samples were prepared to assess the cytotoxic effect of yeast isolates on the chosen cancer cell lines MCF7 (Breast cancer) and IMR32 (Neuroblastoma). Among three samples, two were prepared by cultivating yeast isolates OBS1 and OBS2 separately overnight and one without yeast in YPD broth medium. After overnight incubation, all the three samples were centrifuged to separate yeast cells and supernatants were filtered using membrane filters to obtain clear sterile test samples. Sulforhodamine B (SRB) assay was performed to assess the anticancer property of yeast isolates (Szajewska et al. 2006). Briefly, 10 × 10^3^ cells in 100 μl Dulbecco’s modified Eagle medium (DMEM) per well were seeded in triplicates in 96-well flat-bottom plates and are allowed to adhere overnight. Following this incubation period, the filtrate obtained from cultured broth of yeast isolates OBS1 and OBS2 were added to each of the wells in a volume dependent manner (2–40 µl) and incubated for 48 h. After 48 h, the media was replaced with 100 µl of 10% Trichloroacetic acid for fixation of cells for 1 h at 4 °C and stained with 0.4% sulforhodamine B dissolved in 1% acetic acid. Cells were then washed with 1% acetic acid to remove unbound dye. The protein-bound dye was extracted with 10 mM Tris base to determine the optical density at 510 nm wavelength.

### Antioxidant properties

The yeast autolysate for determination of antioxidant activity was prepared as per the protocol described by Chana et al. (2005). Cell pellets of two active yeast isolates, OBS1 and OBS2 were suspended in 20 ml sterile distilled water and incubated at 25 °C for 72 h. After incubation, the content was centrifuged and the volume of separated supernatant autolysate solution was made up to 500 ml with distilled water. The antioxidant activity of yeast isolates was assayed using phosphomolybdenum method (Kumaran and Karunakaran 2007). The increased absorbance of reaction mixture indicates the increased antioxidant property.

### Statistical analysis

Statistical analysis was carried out for the data using the Statistical Package for the Social Sciences (SPSS), version 12.0 for Windows, for the determination of average and standard deviations. In this analysis, independent variables are temperature, pH, ox-bile, organic acid mixture, gastric enzymes like amylase, trypsin and pepsin and the dependent variables are the percent tolerance of yeast isolates. Significance was determined to be p < 0.01 using two-way analysis of variance (ANOVA), followed by Duncan’s multiple range test.

### Sequence and culture deposition

Sequences were submitted to the NCBI GenBank and acquired accession numbers for both the strains. OBS1: KP998095 and OBS2: KP998094. Based on probiotic and therapeutic properties, yeast OBS2 was selected and deposited into DSMZ culture collection as *S. cerevisiae* DSM 103642.

## Results

### Isolation and associated growth/morphological characteristics of yeast strains

A total of 53 yeast isolates (OBS1-OBS53) were isolated from the nectar of toddy palm collected from different regions of Telangana state of India. The basic idea behind this is to enable isolation of yeast strains from different hostile environments which will facilitate the function of isolated strains with plausible efficient probiotic characteristics.

Fifteen yeast isolates (OBS1-OBS15) were selected for further studies based on their differential growth and colony morphology like margin, elevation, color, type of fermentation and shape.

The yeast isolate OBS12 showed biomass formation upon mannitol supplementation, *S. boulardii* showed biomass formation in Lactose supplemented medium. However, all isolates did not form biomass in ribose, cellulose, starch supplemented media. Except OBS9, OBS2 and OBS4, biomass was observed for all the isolates in galactose supplemented medium (Table 1).

**Table 1:** Sugar assimilation profile of yeast isolates

S. no	Isolate	A	B	C	D	E	F	G	H	I	J	K	L	M
1	*S*. *boulardii*	+	+	+	−	+	−	−	−	+	+	+	+	−
2	OBS1	+	+	+	−	+	−	−	−	+	+	−	+	−
3	OBS2	+	+	+	−	+	−	−	−	+	+	−	−	−
4	OBS3	+	+	+	−	+	−	−	−	+	+	−	+	−
5	OBS4	+	+	−	−	+	−	−	−	+	+	−	−	−
6	OBS5	+	+	+	−	+	−	−	−	+	+	−	+	−
7	OBS6	+	+	+	−	+	−	−	−	+	+	−	+	−
8	OBS7	+	+	+	−	+	−	−	−	+	+	−	+	−
9	OBS9	+	+	+	−	+	−	−	−	+	+	−	−	−
10	OBS10	+	+	+	−	+	−	−	−	+	+	−	+	−
11	OBS12	+	+	+	−	+	+	−	−	+	+	−	+	−
12	OBS13	+	+	+	−	+	−	−	−	+	+	−	+	−
13	OBS14	+	+	+	−	+	−	−	−	+	+	−	+	−

*A* glucose, *B* fructose, *C* maltose, *D* ribose, *E* dextrose, *F* mannitol, *G* cellulose, *H* starch, *I* xylose, *J* sucrose, *K* lactose, *L* galactose, *M* control

(+) indicates presence of biomass and (−) indicates absence of biomass

### Probiotic properties of yeast isolates

#### Antimicrobial drug sensitivity and antagonistic properties

**Fig. 1:**
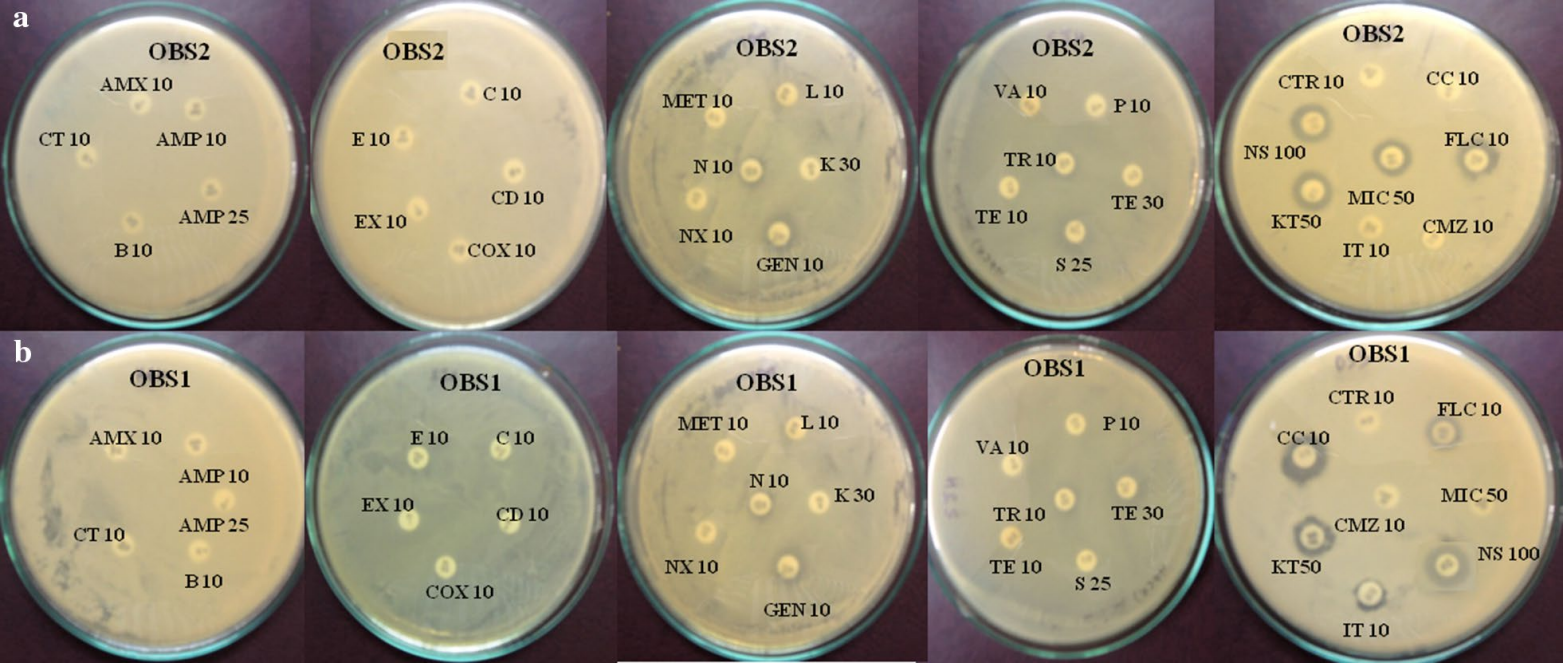
Antimicrobial drug sensitivity of **a** yeast isolate OBS2 and **b** OBS1

Fifteen yeast isolates were tested for antimicrobial drug susceptibility by disc diffusion method. The results of the antimicrobial drug susceptibility of the yeast strains OBS1 and OBS2 against 21 antibacterial and 8 antifungal drugs are summarized in Fig. 1. All the 15 isolates were resistant to the β- lactams cephalosporins i.e. Cefmetazole (30 µg) and Ceftazidime (10 µg); OBS1 and OBS10 were resistant to the cell wall biosynthesis inhibitor and the antifungal agent Clotrimazole (10 µg); *S. boulardii*, OBS4 and OBS10 were resistant to synthetic Triazole antifungal agent Fluconazole (10 µg); *S. boulardii*, OBS1, OBS2, OBS3, OBS5, OBS10, OBS13 and OBS15 were resistant to azole antifungal, Itraconazole (10 µg) and OBS9 was resistant to Ketoconazole (50 µg), Miconazole (50 µg), the azole antifungals and Nystatin, an antimycotic polyene antibiotic (100 µg). The other isolates were sensitive to all the antifungal agents. Yeast isolate OBS2 was sensitive to aminoglycosides-Streptomycin (10 µg) and Gentamicin (10 µg) with 0.1 and 0.2 cm zone of inhibition, respectively whereas OBS1 was sensitive to Gentamicin (10 µg) with 0.2 cm zone of inhibition. All other isolates were resistant to all antibacterial agents and hence did not show any zone of inhibition around the disc.

Based on the results of morphological, cultural and antimicrobial drug sensitivity profiles, 8 isolates (i.e. OBS1, OBS2, OBS4, OBS7, OBS9, OBS11, OBS12 and *S. boulardii*) were selected for further studies.

**Fig. 2:**
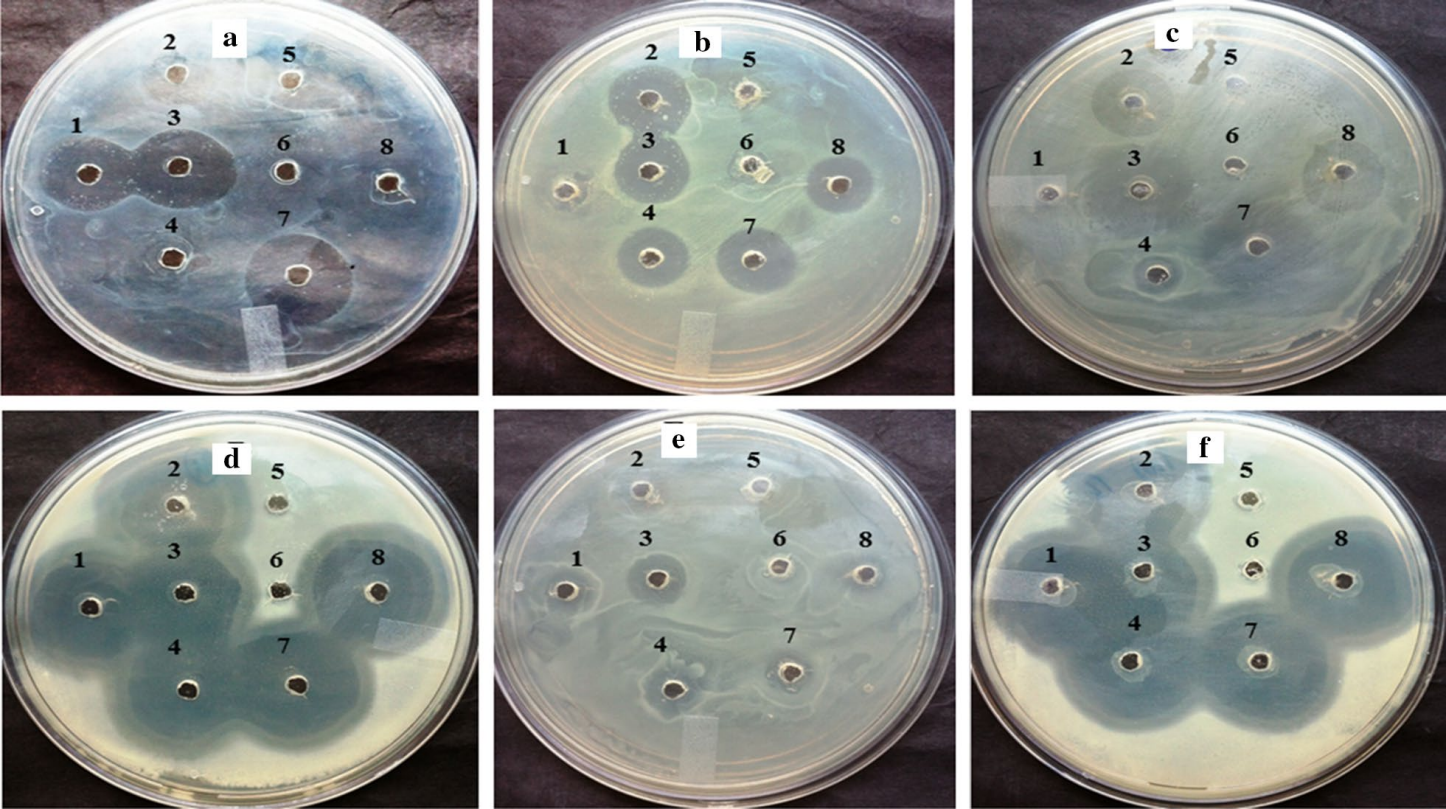
Antagonistic activity of yeast isolates *1* OBS1, *2* OBS2, *3* OBS3, *4* OBS4, *5* OBS7, *6* OBS9, *7* OBS12 and *8 S. boulardii* on human intestinal pathogens **a** *Escherichia coli* 0157:H7, **b** *Pseudomonas aeruginosa*, **c** *Klebsiella pneumonia*, **d** *Staphylococcus aureus*, **e** *Salmonella typhi* and **f** *Salmonella paratyphi*

The results clearly indicate that 12 h grown cultures of all the eight yeast isolates and 24 h grown cultures of OBS7 and OBS9 did not show any antimicrobial/antagonistic activity towards intestinal pathogenic bacteria. Pathogenic *E*. *coli* 0157:H7 was resistant to *S. boulardii* and OBS4 whereas *K. pneumoniae* was resistant to 24 h grown cultures of OBS1. Bacterial cultures like *E. coli 0157:H7*, *P. aeruginosa, K. pneumonia, Staph. aureus, S. typhi* and *S. paratyphi* were sensitive to yeast isolates OBS1 with 0.8, 0.2 0.0, 0.8, 0.3 and 0.8 cm, OBS2 with 0.5, 0.5, 0.6, 0.8, 0.3 and 0.8 cm, OBS3 with 0.8, 0.3, 0.8, 0.6, 0.3 and 0.8 cm, OBS4 with 0.0, 0.3, 0.2, 0.5, 0.3 and 0.8 cm and OBS12 with 1.0, 0.5, 1.0, 1.0, 0.2 and 1.0 cm zone of inhibition, respectively (Fig. 2).

Yeast isolates OBS1, OBS2, OBS3, OBS4, OBS7, OBS9 and OBS12 showed percent cell viability of 78, 85, 81, 78, 28, 35 and 62, respectively when cultured with *E*. *coli* 0150:H5. Similarly the isolates OBS1, OBS2, OBS3, OBS4, OBS7, OBS9 and OBS12 showed percent cell viability of 53, 82, 25, 28, 25, 26 and 73, respectively when cultured with *L. acidophilus*. Whereas *S. boulardii sh*owed 53 percent viability with *E*. *coli* 0150:H5 and 62 percent viability with *L. acidophilus*. In none of the above studies cell viability of neither *E*. *coli* 0150:H5 (86 percent) nor *L. acidophilus* (84 percent) got affected by yeast isolates.

#### Stress tolerance

Tolerance to temperature by yeast isolates OBS2 and OBS9 was significantly (p < 0.01) different from other isolates at 45  and 40 °C, respectively.. Tolerance observed for OBS2 at 45 °C, pH 2, 1 percent ox-bile and 1 percent organic acid mixture were 97.66, 92.66, 92.33 and 91.33 percent, respectively which were significantly (p < 0.01) different from other yeast isolates (Table 2).

**Table 2:** Percent tolerance of yeast isolates at different temperature, pH, Ox-bile and organic acid mixture concentrations

Sl. no.	Parameters	Units	Percent tolerance of yeast isolates								SEM
			*S*. *boulardii*	OBS1	OBS2	OBS3	OBS4	OBS7	OBS9	OBS12	
1	Temp (°C)	30	99.33 ± 0.47^b^	99.00 ± 0.40^c^	99.66 ± 0.23^a^	99.00 ± 0.40	98.66 ± 0.47	99.33 ± 0.23^b^	98.66 ± 0.47	99.33 ± 0.23^b^	0.36
		37	99.00 ± 0.40^a^	98.66 ± 0.47^b^	99.00 ± 0.40^a^	99.00 ± 0.40^a^	97.00 ± 1.00	96.66 ± 1.60	97.66 ± 0.84^c^	96.66 ± 1.17	0.81
		40	98.33 ± 0.62^c^	91.33 ± 0.47	99.00 ± 0.40^b^	96.66 ± 0.62	94.66 ± 0.23	97.00 ± 0.70	99.33 ± 0.94^a^	92.66 ± 0.47	0.56
		45	94.66 ± 0.23^b^	83.33 ± 0.23	97.66 ± 0.23^a^	91.33 ± 0.47^c^	91.00 ± 0.40	90.33 ± 0.23	90.66 ± 0.23	86.00 ± 0.35	0.30
2	pH	2.0	89.66 ± 0.23^b^	82.00 ± 0.81^c^	92.66 ± 0.47^a^	79.33 ± 0.23	80.66 ± 0.23	84.66 ± 0.23	79.66 ± 0.23	75.66 ± 0.23	0.33
		2.5	91.33 ± 0.47^b^	84.66 ± 0.23	94.66 ± 0.23^a^	81.33 ± 0.47	83.33 ± 0.47	88.66 ± 0.47^c^	83.33 ± 0.47	79.66 ± 0.23	0.38
		3.0	94.66 ± 0.23^b^	91.00 ± 0.40^c^	97.00 ± 0.40^a^	84.00 ± 0.81	87.33 ± 0.84	91.00 ± 0.40	85.66 ± 0.23	87.00 ± 0.40	0.47
		3.5	94.66 ± 0.23^b^	91.00 ± 0.40^c^	97.00 ± 0.40^a^	84.00 ± 0.81	87.33 ± 0.84	91.00 ± 0.40	85.66 ± 0.23	87.00 ± 0.40	0.47
3	Ox-Bile (%)	0.25	98.66 ± 0.47^b^	98.66 ± 0.62^b^	99.66 ± 0.23^a^	97.66 ± 1.02	98.66 ± 0.62^b^	97.00 ± 1.08	98.00 ± 0.81^c^	98.00 ± 0.81^c^	0.71
		0.50	97.66 ± 0.23^b^	97.00 ± 0.40^c^	99.66 ± 0.23^a^	96.33 ± 0.47	95.66 ± 0.23	94.66 ± 0.23	94.66 ± 0.23	96.00 ± 0.40	0.33
		0.75	95.33 ± 0.23^b^	92.66 ± 0.47	97.66 ± 0.23^a^	93.66 ± 0.23^c^	91.66 ± 0.23	90.66 ± 0.23	91.33 ± 0.47	91.33 ± 0.47	0.32
		1.0	90.66 ± 0.47^b^	88.66 ± 0.23	92.33 ± 0.23^a^	89.66 ± 0.23^c^	84.66 ± 0.23	85.66 ± 0.23	91.33 ± 0.47	87.33 ± 0.47	0.29
4	Organic acid mixture (%)	0.25	97.66 ± 1.02	97.00 ± 1.08	99.33 ± 0.23^a^	95.66 ± 1.54	97.33 ± 0.94	98.33 ± 0.62^b^	98.00 ± 0.81^c^	95.33 ± 0.69	0.99
		0.50	96.33 ± 0.62^b^	95.00 ± 0.40^c^	99.33 ± 0.23^a^	90.33 ± 0.23	95.00 ± 0.23^c^	91.33 ± 0.62	93.66 ± 0.62	90.33 ± 0.62	0.45
		0.75	91.33 ± 0.47^b^	90.66 ± 0.23^c^	94.00 ± 0.40^a^	85.66 ± 0.47	91.33 ± 0.47^b^	88.66 ± 0.23	88.00 ± 0.40	86.00 ± 0.40	0.38
		1.0	85.33 ± 0.23^b^	84.33 ± 0.23^c^	91.33 ± 0.47^a^	80.33 ± 0.62	80.33 ± 0.47	82.66 ± 0.23	85.33 ± 0.23^b^	81.66 ± 0.62	0.39

^a, b, c^Values with different superscripts in a row differ significantly (p < 0.01)

The tolerance ability of yeast isolates to various digestive enzymes such as amylase, pepsin and trypsin was observed and OBS1, OBS2 and *S. boulardii* showed better tolerance compared to other isolates. However, the percent tolerance observed for OBS2 at 350 IU ml^−1^ amylase, 0.75 mg ml^−1^ trypsin and 6.0 g l^−1^ pepsin was 89.33, 99.33 and 96.66, respectively and significantly (p < 0.01) different from other isolates (Table 3).

**Table 3:** Percent tolerance of yeast isolates at different concentration of human gastric enzymes

Sl. no.	Gastric enzymes	Units	Percent tolerance of yeast isolates								SEM
			*S*. *boulardii*	OBS1	OBS2	OBS3	OBS4	OBS7	OBS9	OBS12	
1	Amylase (IU)	250	95.66 ± 0.84^b^	93.33 ± 0.23	98.66 ± 0.62^a^	91.33 ± 0.47	95.66 ± 0.23^b^	92.66 ± 0.62	94.66 ± 0.62^c^	93.66 ± 1.02	0.58
		300	91.33 ± 0.47^b^	87.66 ± 0.62	96.66 ± 0.47^a^	84.33 ± 0.47	89.66 ± 0.62^c^	87.00 ± 0.81	88.33 ± 0.62	85.66 ± 0.84	0.61
		350	86.33 ± 0.62^b^	81.00 ± 0.40	89.33 ± 0.23^a^	78.66 ± 0.47	84.66 ± 0.62^c^	80.33 ± 0.84	84.33 ± 0.62	78.33 ± 1.02	0.6
2	Trypsin (mg/ml)	0.25	99.33 ± 0.23^a^	98.33 ± 0.62^c^	99.33 ± 0.23^a^	97.66 ± 1.02	97.33 ± 0.94	98.00 ± 0.70	98.66 ± 0.47^b^	98.66 ± 0.62^b^	0.6
		0.50	99.66 ± 0.23^a^	96.66 ± 0.47^c^	99.66 ± 0.23^a^	95.66 ± 0.23	95.66 ± 0.84	95.33 ± 0.23	98.33 ± 0.62^b^	96.00 ± 0.40	0.41
		0.75	96.66 ± 0.62^b^	92.66 ± 1.02	99.33 ± 0.23^a^	90.66 ± 0.47	90.33 ± 0.47	90.66 ± 0.23	95.33 ± 0.23^c^	90.66 ± 0.47	0.47
3	Pepsin (g/l)	2	98.33 ± 0.23^b^	96.33 ± 0.62^c^	98.66 ± 0.62^a^	96.33 ± 0.62^c^	96.33 ± 0.47^c^	95.33 ± 0.47	96.00 ± 0.40	95.33 ± 0.47	0.65
		4	96.66 ± 0.47^b^	95.33 ± 0.62	98.33 ± 0.62^a^	95.00 ± 0.70	95.66 ± 1.31^c^	94.66 ± 0.62	94.33 ± 1.02	95.00 ± 1.22	0.68
		6	93.66 ± 0.62	94.66 ± 0.94^b^	96.66 ± 0.47^a^	94.33 ± 0.47^c^	94.33 ± 0.23^c^	94.33 ± 0.84^c^	93.66 ± 0.62	94.66 ± 0.47^b^	0.58

^a,b,c^Values with different superscripts in a row differ significantly (p < 0.01)

Among the 7 yeast isolates, OBS1 and OBS2 tolerate extreme conditions of the gastrointestinal tract like temperature, low pH, organic acids and digestive enzymes, i.e. pepsin and amylase in the stomach and trypsin and bile in the upper intestine.

#### Pathogenicity of yeast isolates

**Fig. 3:**
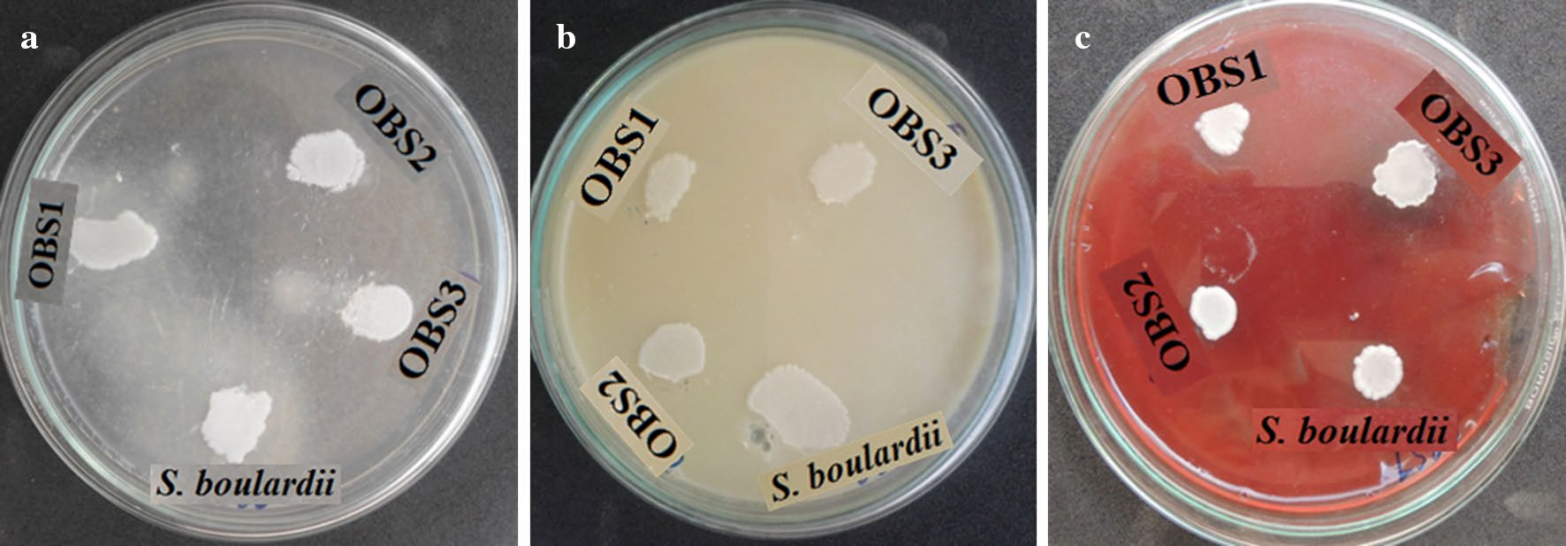
Pathogenicity of yeast isolates and commercial probiotic *S. boulardii*: **a** protease activity, **b** phospholipase activity and **c** haemolytic activity

**Fig. 4:**
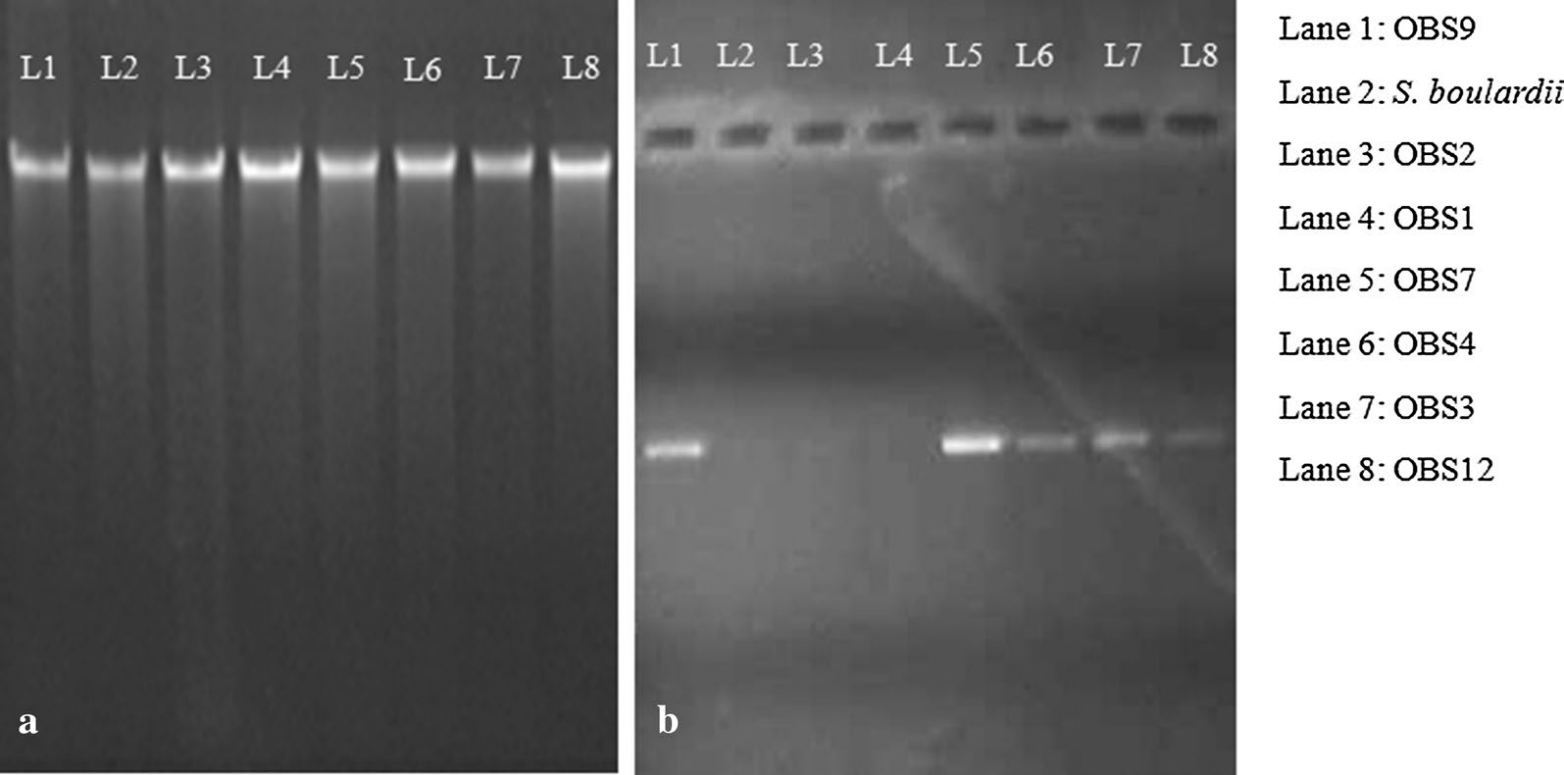
Pathogenicity of yeast isolates based on gene amplification: **a** genomic DNA of the yeast isolates and **b** amplicons of yeast isolates

The seven yeast isolates did not show any zone of hydrolysis around the colony on specific media indicating that none of the isolates have protease, phospholipase and haemolytic activity (Fig. 3). Further, except OBS1, OBS2 and *S. boulardii* the template DNA was amplified with primers of human pathogenic yeast *C. albicans* and hence were considered as pathogens. The yeast isolates OBS1 and OBS2 were considered as non pathogens and selected for further studies (Fig. 4).

#### Cell adhesion

Successful probiotic microorganisms are able to colonize by adhering to the intestinal mucosa at least temporarily. Therefore, we determined the adhesion ability of yeast isolates by following the protocol as mentioned in materials and methods. The mean number of yeast cells was counted and the adherence score for each of the yeast isolate was determined on the basis of size of the inoculum. Among all the yeast isolates, *S. boulardii,* OBS1 and OBS2 expressed a strong adhesive ability of more than 40 yeast cells adhering to the monolayer.

### Molecular characterization

**Fig. 5:**
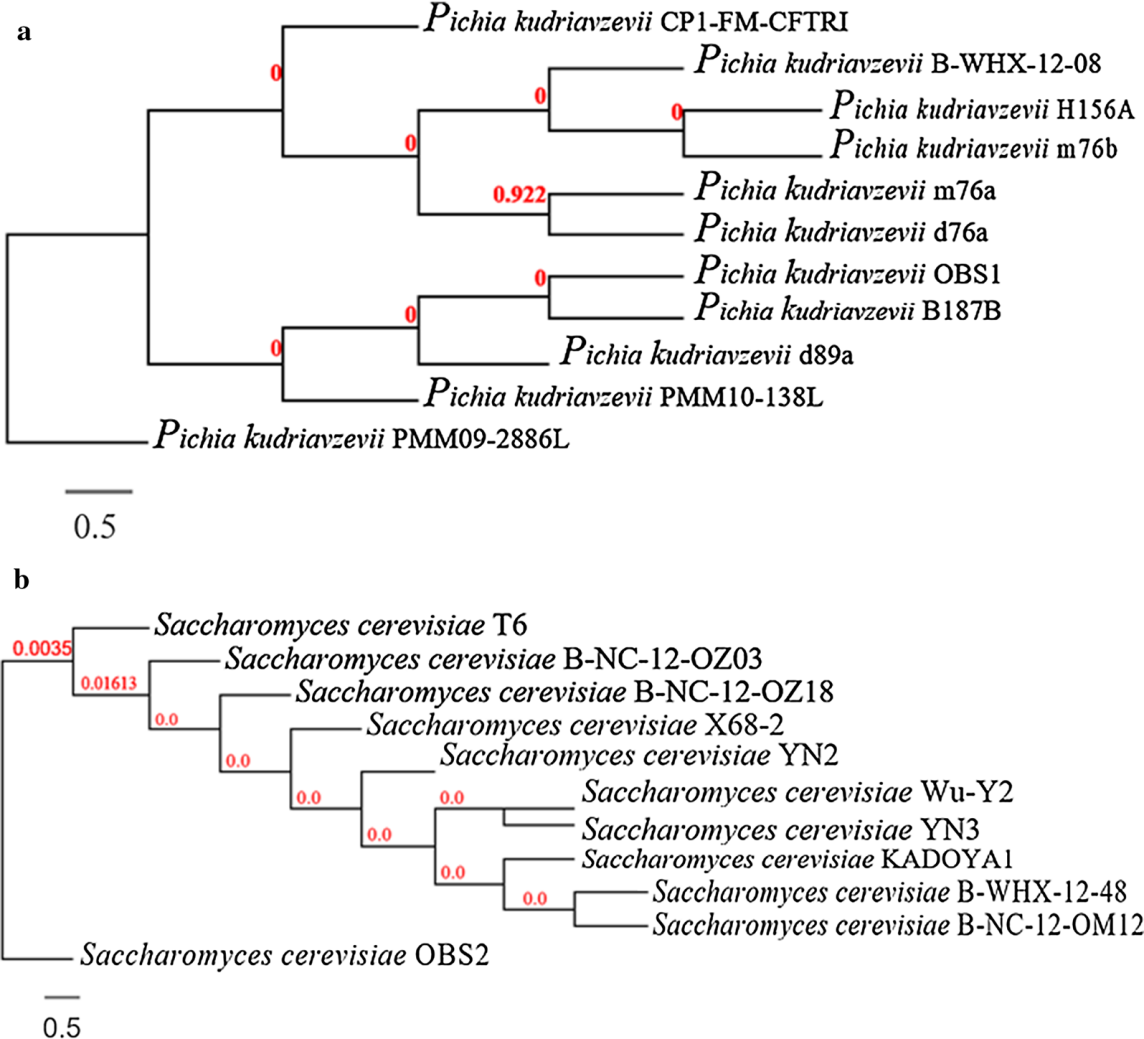
Phylogenetic dendrogram of yeast isolates **a** *P*. *kudriavzevii* OBS1 and **b** *S*. *cerevisiae* OBS2

The yeast isolate OBS2 has 96% identity and query covers 97% and E value is zero with *Saccharomyces cerevisiae* (Fig. 5b), whereas OBS1 has 100% identity and query covers 68% and E value is zero with *Pichia kudriavzevii* (Fig. 5a). The 1370 and 822 base length gene sequences of OBS1 and OBS2 isolates were deposited in the NCBI-GenBank data library and acquired accession number KP998094 for *S. cerevisiae* OBS2 and KP998095 for *P*. *kudriavzevii* OBS1. These two yeast isolates were used for assessment of therapeutic properties.

### Therapeutic properties

**Fig. 6:**
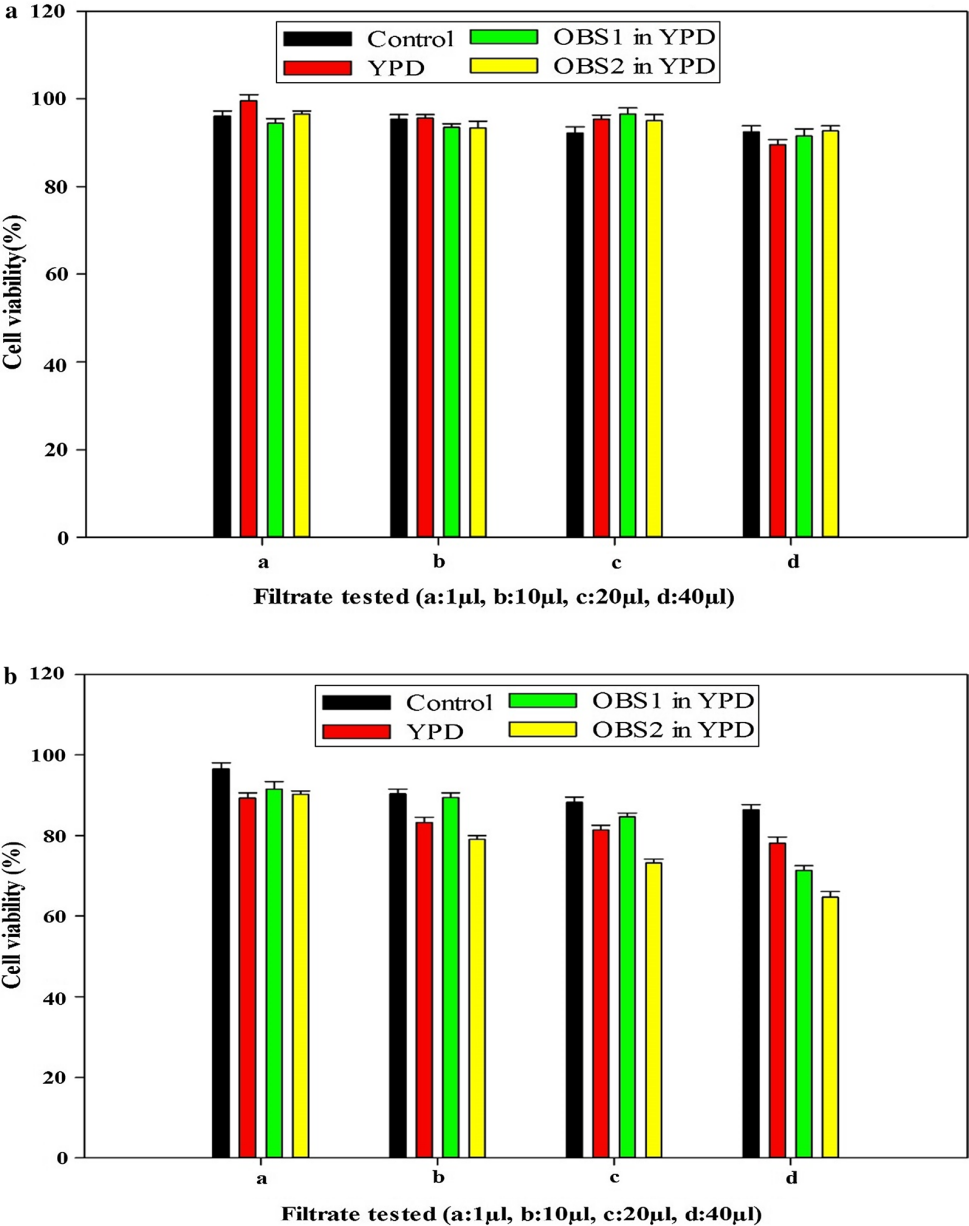
Effect of yeast isolates on viability of **a** breast cancer cell line MCF7 and **b** neuroblastoma cell line IMR32

Out of the two tested yeast samples, sample pertaining to yeast isolate OBS2 resulted in a slight reduction in percentage cell viability of neuroblastoma cell line IMR32 (Fig. 6b) but no reduction in percentage cell viability of breast cancer cell line MCF7 (Fig. 6a) when compared to the control and YPD broth.

The autolysate of yeast isolate OBS1 has shown decreased absorbance i.e. 1.799, 1.685 and 1.542 at 695 nm with the increased concentration of autolysate i.e. 0.1, 0.2 and 0.3 ml and it was higher than the positive control where it showed 1.642 at 0.3 ml concentration of ascorbic acid. This indicates no antioxidant property. Whereas the autolysate of yeast isolate OBS2 showed increased absorbance i.e. 1.344, 1.454 and 1.528 at 695 nm with the increased concentration of autolysate i.e. 0.1, 0.2 and 0.3 ml and it was lesser than the positive control indicates it has antioxidant property.

## Discussion

Culturally and morphologically characterized yeast isolates were resistant to majority of the antibacterial drugs, because of their cell wall composition which differs with prokaryotes (Czerucka et al. 2007) and most of the isolates were sensitive towards maximum antifungal drugs (Mashad et al. 2012).

Antagonistic activity was observed for 24 h grown but not 12 h grown yeast isolates against human intestinal pathogens. When they cultured in a mixed population of intestinal commensals, these isolates showed maximum co-cultural activity (Izadnia et al. 1998; Filho-Lima et al. 2000; Czerucka and Rampal 2002). Antagonistic activity of yeast isolates may be due to the production of antimicrobial metabolites (Hatoum et al. 2012; Rao et al. 2009). The present findings of antagonistic and co-cultural properties of yeast isolates are in accordance with Verna and Lucak (2010). The yeast isolates OBS1 and OBS2 were resilient to gastrointestinal parameters is acceptable, when compared with the findings of Kuhle et al. (2005), Bhima et al. (2010), Rajkowska and Kunicka-Styczynska (2010).

The yeast isolates OBS1 and OBS2 did not show any pathogenic properties and hence confirmed as non-pathogens and which is in accordance with the reports of Koehn (1982), Manns et al. (1994), Luo et al. (2001) and Lechuga et al. (2016). Similarly, these isolates have shown strong adhesive property to human intestinal cells (Caco-2). This finding is in accordance with Zivkovic et al. (2015), where the probiotic organism adhesion is more than 40 cells to intestinal cells (Caco-2).

Yeast isolates OBS1 and OBS2 were characterized based on phylogenetic analysis as *P. kudriavzevii and S. cerevisiae*. Among the two yeast isolates, OBS2 has a little cytotoxic effect on Neuroblastoma cell line IMR32 which means continuous use of the isolate OBS2 as probiotics may help in curing the incidence of neuroblastoma cancer. On the other hand the same isolate OBS2 has an antioxidant property and by increased cell concentration may improve its probiotic efficiency, which is in agreement with the findings of Naylin et al. (2005) and Oh et al. (2002).

Based on resilience properties to gastrointestinal parameters, antimicrobial agents sensitivity, antagonistic activity, pathogenicity, co-cultural activity, adherence ability to intestinal epithelial cells, 2 yeast isolates were selected and characterized as *P. kudriavzevii* OBS1 and *S. cerevisiae* OBS2. Of the two isolates *S. cerevisiae* OBS2 has cytotoxic effect on cancer cells and antioxidant activity hence can be used for human probiotic and therapeutic applications.

## Abbreviations

QPS: qualified presumption of safety
YPD: yeast extract peptone dextrose
YNB: yeast nitrogen base
CFU: colony forming units
PBS: phosphate buffered saline
PCR: polymerase chain reaction
NCCS: National Centre for Cell Science
MEM: Eagle’s minimal essential medium
ITS: internal transcribed spacer
BLAST: basic local alignment search tool
NCBI: national center for biotechnology information
DMEM: Dulbecco’s modified Eagle medium
ANOVA: analysis of variance
DSMZ: Deutsche Sammlung von Mikroorganismen und Zellkulturen GmbH
SRB: sulforhodamine B
